# Chocolate Consumption and Sex-Interest

**DOI:** 10.7759/cureus.13310

**Published:** 2021-02-12

**Authors:** Beatrice A Golomb, Brinton K Berg

**Affiliations:** 1 Medicine, University of California, San Diego, USA

**Keywords:** sex-interest, chocolate, testosterone, female diet, libido, cross-sectional study, sex differences

## Abstract

Media and popular literature link chocolate and sex-interest in women, but there is little research examining their association. This cross-sectional analysis sought to address this gap by assessing the relation of chocolate-consumption frequency to self-rated interest in sex. Seven-hundred twenty-three (723) Southern California men and women, age >20, completed surveys providing chocolate-consumption frequency (Choc0, x/week) and interest in sex (rated 0-10).

Regression (robust standard errors) examined the relationship of chocolate-consumption frequency (Choc0, x/week) to sex-interest, adjusted for potential confounders. Tests for gender and age interactions guided gender- and age-stratified analyses. The mean sex-interest was 7.0±3.0 overall; 5.7±3.1 in women and 7.4±2.8 in men. The reported chocolate frequency was 2.0±2.5x/week overall; 2.5±2.8x/week in women and 1.8±2.4x/week in men. Those who ate chocolate more frequently reported lower interest in sex. Significance was sustained with an adjustment: per-time-per-week chocolate was eaten, β=-0.11(SE=0.050), p=0.02. The gender interaction was significant (p=0.03). The gender-stratified analysis showed the effect was driven by the much stronger relation in women: full model, per time-per-week chocolate consumed, β=-0.26(SE=0.08), p=0.002. Chocolate-consumption frequency was the strongest assessed predictor of sex-interest in women. A relationship was not observed in men, though a trend was present in younger men.

Women who ate chocolate more frequently reported less interest in sex, a finding not explained by assessed potential confounders. Popular portrayals in which chocolate is represented as substituting for sex and “satisfying” the need for sex in women represent one possible explanation for these findings.

## Introduction

Chocolate is an iconic Valentine gift, with candy (75% chocolate [1]) accounting for ~$2 billion (US) in Valentine-related sales annually [2]. Per CNN, 85% of men and women say sex is an important part of Valentine’s Day [2] and 32% of Americans said that they were likely to have sex on Valentine’s Day [1]. In the West, Valentine’s chocolate is most typically gifted by men (bearing the statistically greater sex drive [3-4] to women. (Who gifts Valentine’s chocolate to whom is reportedly reversed in South Korea [2].)

Scientific themes suggest a biochemical link. Phenylethylamine in chocolate [5] has been referred to as the “love” chemical [6]. News stories report that compounds in chocolate stimulate the chemical pleasures associated with sex [6], buttressed by scientific articles reporting that compounds in chocolate lead to the release of serotonin and dopamine, pleasure- and reward-signaling chemicals [7]. In line with this, popular references to chocolate in lieu of or in substitution for sex are legion. An Internet search of chocolate images shows humorous portrayals that “yes, size matters” (depicting a woman eating a large chocolate bar); stating “because chocolate can’t get you pregnant;” representing chocolate as the perfect Valentine date (it never disappoints); or stating “chocolate is like sex, but you don’t have to shave your legs” [8-10].

Considering the above, chocolate consumption might be tied to increased sex-interest - or diminished (or neither). Chocolate might be postulated to spur interest in sex, by simulating love and stimulating the chemicals allied with it - with love, in turn, reported to increase interest in sex among women [11]. Alternatively - analogous to methadone relieving the drive for heroin use - chocolate, by replicating the satisfactions and pleasures of sex, may obviate the need for it.

In short, chocolate might stimulate sex - or simulate it.

Considering the fertile discourse linking sex and chocolate, the dearth of science to inform this discourse is striking. We sought to take an initial step toward redressing this scientific void. We capitalized on data previously collected, to assess the cross-sectional relationship of chocolate-consumption frequency to interest in sex.

Since we will concurrently address study participants’ “sex” (the male-female distinction) and “sex” (the activity), we will employ the term “gender” for male vs female to avert confusion. (We recognize that this usage is abjured: Per the New York Times, “nouns have gender; people, bless their hearts, have sex” [12].)

## Materials and methods

Participants and design

One-thousand eighteen (1018) community-dwelling adults from southern California, without known diabetes, cardiovascular disease, or extremes of high or low-density lipoprotein (LDL) cholesterol (<115mg/dL or >190mg/dL), were screened for participation in a study (examining the effects of lowering cholesterol) that sampled broadly across the adult age range. The sex-interest inquiry was an added partway into the study/screening process so was not available for all participants: 723 completed the sex-interest question, including 559 men and 164 women, age 20 to 85 (participants not completing this question were not included in the analysis). All participants gave written IRB-approved informed consent (“consenting adults”). Assessments reported here were undertaken prior to any receipt of treatment. Because of the potential to be placed on a drug, women of childbearing potential were excluded, creating an expected male preponderance.

Sex-interest was not a prespecified outcome, but a stated tertiary intent of the study was to capitalize on study data for a cross-sectional examination of predictors of behaviors and outcomes. The present analysis is cross-sectional.

Measurements

For sex-interest, participants were asked to rate "interest in sex" (past two weeks), on a scale of 0-10, with 0 defined as "not present" and 10 defined as "maximally present." For chocolate frequency, participants were asked "how many times a week do you consume chocolate?" Multiple forms of evidence support the validity of these measures [13].

Covariates considered for adjustment included variables that bear literature-documented relations to sex-interest (and to chocolate), so might plausibly serve as confounders.

Calories/day consumed (derived from the Fred Hutchinson Food Frequency Questionnaire) was included due to a reported relation between calorie restriction and reduced interest in sex [14] - and a reported relation of chocolate consumption to calorie consumption [15]. Mood via the Center for Epidemiological Studies-Depression scale (CES-D) was included as mood relates to an interest in sex [16], as well as to chocolate consumption (determined in this sample) [17]. The measured serum testosterone level was included since testosterone relates to sex-interest [18-19]. Blood pressure was included since low/lowered blood pressure can be adverse to sexual function, which could influence sex-interest [20].

Ethnicity was also included as a covariate by convention, although it did not meet the criteria as a potential confounder. Assessments with and without ethnicity were performed, as below. For ethnicity, participants selected from among provided ethnicity classifications (White, not Latino; Latino/Hispanic; African American; Asian/Pacific Islander; Native American; Other) and were instructed to select the most appropriate.

Continuous variables were not binned for analysis except as expressly stated (e.g., in an exploratory assessment stratified by age).

Analysis

The characteristics of respondents were assessed (mean ± SD, or percent for categorical variables) for the combined-gender sample and for men and women separately.

Variables selected as regression covariates were evaluated in this sample against sex-interest and chocolate frequency to affirm relevance empirically in the dataset. (This simultaneously provides internal evidence bearing on the validity of the sex-interest outcome.)

LDL-cholesterol and glucose were also assessed against sex-interest and chocolate on an exploratory basis to assess whether these variables, which influenced study eligibility, met criteria as potential confounders.

Correlation was used to examine the raw cross-sectional relationship of chocolate-consumption frequency with sex-interest.

Regression analysis with robust (heteroskedasticity-independent) standard errors [21] re-examined the relationship between chocolate frequency and interest in sex. This was evaluated in an unadjusted regression, as well as a “limited” model adjusted for age, gender, and ethnicity, and in a “full” model also adjusting for blood pressure (mmHg), mood (CES-D), calories/day, and testosterone (ng/mL). Regression (limited and full models) was performed with and without adjustment for ethnicity.

We assessed the significance of a gender-by-chocolate interaction term added to the full model to determine whether a gender-stratified analysis was merited. An age-interaction term was also assessed (splitting the sample at age 55, approximating the mean age of the sample).

Analyses used Stata versions 8.0 and 12.0 (StataCorp, College Station, TX). Two-sided p-values < 0.05 designated statistical significance.

## Results

Sample characteristics are shown in Table 1. Mean sex-interest (rated 0-10) was 7.0 overall, with mean chocolate frequency (x/week) 2.0 overall. Mean sex-interest was 5.7 in women and 7.4 in men, with the male-female difference significant on t-test (p<0.001) and in regression adjusted for age (p<0.001). Mean chocolate-frequency was 2.5x/week in women and 1.8x/week in men; the difference was also significant on t-test (p=0.003) and in regression adjusted for age (p=0.02). (The difference in p-values on t-test vs regression is partly attributable to the use of robust standard errors in the regression. In absence of this, the p-value with regression analysis is p=0.007.)

**Table 1:** Participant characteristics (mean (SD)*) *Except items designated as %. †Percent for whom the variable was available. BP=blood pressure; CES-D=Center for Epidemiological Studies-Depression scale; LDL=low-density lipoprotein cholesterol; N=number SI conversion factors: To convert testosterone to nmol/L, multiply values by 3.47. To convert cholesterol to mmol/L, multiply values by 0.0259. To convert glucose to mmol/L, multiply values by 0.0555.

	All	%†	Women	Men
	N =723	--	N=164	N=559
Sex-interest	7.0 (3.0)	--	5.7 (3.1)	7.4 (2.8)
Chocolate (x/week)	2.0 (2.5)	100	2.5 (2.8)	1.8 (2.4)
Age (years)	56 (12)	100	60 (9.3)	54 (13)
Gender (% Male)	77	100	--	--
Ethnicity (% Caucasian)	81	100	84	80
Calories/day	1734 (824)	93	1508 (624)	1800 (863)
Testosterone (ng/ml)	3.5 (2.2)	95	0.38 (0.45)	4.4 (1.6)
Diastolic BP (mm Hg)	75 (8.8)	96	73 (9.5)	75 (8.6)
Systolic BP (mm Hg)	127 (14)	96	125 (16)	127 (14)
Mood (CES-D)	8.1 (7.5)	99	8.4 (7.9)	8.0 (7.3)
LDL-cholesterol (mg/dL)	150 (26)	99	153 (28)	149 (25)
Fasting glucose (mg/dL)	90 (9.1)	100	88 (9.0)	91 (9.1)

Greater chocolate-consumption frequency correlated significantly to lesser self-rated interest in sex in the combined-gender sample, r=-0.12 (p=0.001).

Covariates considered for adjustment (except ethnicity) were affirmed to relate, as trend or effect, to sex-interest (each p<0.15 in the expected direction). All but blood pressure (and ethnicity) also met this threshold for significance vis-a-vis chocolate frequency, validating the importance of inclusion in the regression. Other examined variables bore no trend in relation to sex-interest (p>0.29 for each), supporting the adjustment choices.

Age, sex, and ethnicity were available for all participants; the food frequency questionnaire, providing calories, was completed and returned by 93%; testosterone levels, measurement of which depended on sample adequacy after other assessments [22], was available in 95%; blood pressure assessment was completed in 96% and mood assessment (CES-D) was completed by 99% of participants. Missing values were not imputed.

Regression results with robust standard errors are shown in Table 2. As evident, the significance of the inverse relation of chocolate frequency to sex-interest was reproduced with regression, and sustained, though attenuated, in the limited and full adjustment models. (Values were closely similar without the inclusion of ethnicity in each model: coefficients were unchanged: p-values were 0.002 (unadjusted), 0.02 (limited), and 0.02 (full).)

**Table 2:** Relation of chocolate consumption frequency to interest in sex* *Regression analyses with robust standard errors. †The coefficient and significance were unaffected by the use of systolic vs diastolic blood pressure. β=regression coefficient; CI=confidence interval; N=number; SE=standard error.

Model	Adjusted variables	All (N= 722)		
		β (SE)	95%CI	P
Unadjusted	None	-0.14 (0.05)	-0.23, -0.06	0.002
Limited	Age, gender, ethnicity	-0.10 (0.04)	-0.18, -0.02	0.02
Full	Age, gender, ethnicity, blood pressure†, mood, calories, testosterone	-0.11 (0.05)	-0.21, -0.02	0.02

A gender-interaction term added to the full model was significant. Chocolate frequency x male gender: β=0.21(SE=0.10), p=0.03. (Magnitude and significance of the chocolate-frequency predictor were strengthened after the addition of this interaction term to the full model: chocolate-frequency coefficient β=-0.26(SE 0.08), p=0.002.) This supported the performance of gender-stratified analyses.

In women, the correlation of chocolate frequency to sex-interest was r=-0.17 (p=0.03). Of note, for women, chocolate frequency was the only assessed variable to correlate significantly with sex-interest. Among men, the correlation of chocolate frequency to sex-interest was smaller and nonsignificant, r=-0.07 (p=0.11).

Table 3 shows the results of the gender-stratified regression analyses. These affirm that significance was driven disproportionately by findings in women, despite their smaller representation in the sample.

**Table 3:** Relation of chocolate-consumption frequency to interest in sex* stratified by gender – with and without ethnicity adjustment *Regression analyses with robust standard errors. †Limited model adjusts for: Age, ethnicity. ‡Full model adjusts for: Age, ethnicity, blood pressure, mood, calories, and testosterone. (Gender adjustment not required in the gender-stratified analysis.) β=regression coefficient; CI=confidence interval; N=number; SE=standard error

With Ethnicity Adjustment						
Model	Women (N= 164)			Men (N=558)		
	β (SE)	95%CI	P	β (SE)	95%CI	P
Unadjusted	-0.19 (0.09)	-0.36, -0.02	0.03	-0.08 (0.05)	-0.18, 0.01	0.09
Limited†	-0.19 (0.08)	-0.35, -0.02	0.03	-0.06 (0.04)	-0.15, 0.02	0.15
Full‡	-0.26 (0.08)	-0.43, -0.10	0.002	-0.04 (0.06)	-0.15, 0.07	0.52
Without Ethnicity Adjustment						
Model	Women (N= 164)			Men (N=558)		
	β (SE)	95%CI	P	β (SE)	95%CI	P
Unadjusted	-0.19 (0.09)	-0.36, -0.02	0.03	-0.08 (0.05)	-0.18, 0.01	0.09
Limited†	-0.19 (0.08)	-0.35, -0.02	0.03	-0.06 (0.04)	-0.15, 0.03	0.17
Full‡	-0.26 (0.08)	-0.43, -0.10	0.002	-0.03 (0.06)	-0.14, 0.07	0.54

For women, the relation of chocolate frequency to sex-interest was sustained with adjustment for age, sex, and ethnicity. Moreover, additional adjustments further strengthened (vs attenuated) significance. In adjusted models (as in comparisons among correlations), chocolate frequency remained the most significant among assessed predictors of sex-interest in women.

In contrast, in men assessed separately, chocolate frequency did not significantly predict sex-interest. P-values in men increased further (i.e., significance was further reduced) with more complete adjustment.

An age x chocolate interaction term (added to the full-adjustment model) was not significant in women (p=0.33) but was borderline significant in men (thresholding for each based on the mean age of that sex - ~age 55 for men: β=0.17(SE=0.10), p=0.09). Stratifying analysis at age 55, greater chocolate frequency negatively predicted sex-interest in younger men, under age 55 years, in unadjusted and limited adjustment models (See Table 4). A trend remained present (p<0.1), but significance was lost in the full model.

**Table 4:** Men stratified by age* – with and without ethnicity adjustment *Regression analyses with robust standard errors β=regression coefficient; CI=confidence interval; SE=standard error

With Ethnicity Adjustment						
Model	Age <=55			Age >55		
	β (SE)	95%CI	P	β (SE)	95%CI	P
Unadjusted	-0.12 (0.06)	-0.24, -0.005	0.04	-0.04 (0.07)	-0.18, 0.10	0.59
Limited: Age, ethnicity	-0.12 (0.06)	-0.24, -0.007	0.04	+0.001 (0.07)	-0.13, 0.13	0.99
Full: Age, ethnicity, blood pressure, mood, calories, testosterone	-0.13 (0.08)	-0.29, 0.02	0.09	+0.08 (0.08)	-0.09, 0.24	0.37
Without Ethnicity Adjustment						
Model	Age <=55			Age >55		
	β (SE)	95%CI	P	β (SE)	95%CI	P
Unadjusted	-0.12 (0.06)	-0.24, -0.005	0.04	-0.04 (0.07)	-0.18, 0.10	0.59
Limited: Age	-0.12 (0.06)	-0.23, -0.0003	0.049	+0.0007 (0.07)	-0.13, 0.13	0.99
Full: Age, blood pressure, mood, calories, testosterone	-0.13 (0.08)	-0.28, 0.02	0.10	+0.08 (0.08)	-0.08, 0.24	0.35

Exploratory analyses in women by decade - based, however, on small numbers so with low confidence - suggested the possibility of nonlinear effect modification by age: associations were strongest in the youngest and oldest women (age under 50, and over 70), with coefficients (full model) of -0.38 for the youngest group and -0.78 for the oldest (recognizing that healthy participant bias is magnified in the oldest ages [23]).

## Discussion

Women who ate chocolate more frequently reported significantly less interest in sex. A qualitatively similar finding was present for analysis of combined men and women. However, the finding was particularly strong among women, and separately significant for women, for whom chocolate-consumption frequency was, indeed, the strongest assessed predictor of sex-interest. On exploratory analysis, younger adult men (under age 55) contributed somewhat to the relationship in the combined-sex sample, but the relationship of more frequent chocolate consumption to lesser sex-interest in younger men was materially weaker than the relationship in women, and the significance of the finding was attenuated with adjustments (vs strengthened in women). Older men did not share this relationship.

Fit with literature

Against a surfeit of popular allusions to a link between sex and chocolate, few studies appear to have sought to empirically assess the relation of chocolate consumption to interest in sex. We identified only one prior study that addressed something nominally similar - assessing prediction by a chocolate measure against an index of “sexual desire” in a convenience sample of women in Northern Italy [24]. Many features of the study affect statistical power and ability to see a relationship [24]: chocolate-consumption frequency was binarized (daily - yes or no) so that those eating chocolate 6x/week are categorized with those eating it never. That study’s “age-adjusted” analysis showed no significant relationship. (In fact, in their sample, younger age was associated both with more “sexual desire” and more daily chocolate consumption, producing a spurious positive association that was obviated with age-adjustment.) However, the study involved a smaller sample, and the binarized chocolate-frequency measure is expected to lose statistical benefits relative to a more continuous analysis approach. That study also did not assess, so could not adjust for, other potential confounders. Applying to our data an approximation to their analysis approach - by binarizing our chocolate measure as they did for comparison - yields their finding of a nonsignificant age-adjusted relationship in women, consistent with the expected loss of important information and statistical power by dichotomizing a more continuous predictor. Since reproducing their analysis decision - one that is associated with the expected loss of power - reproduces their null finding, that finding does not challenge our own.

Though the scientific context for our finding is limited, nonscientific representations relevant to our finding are rife and motivated the present study. Internet quote sites provide these examples: "It's not that chocolates are a substitute for love. Love is a substitute for chocolate. Chocolate is, let's face it, far more reliable than a man." - Miranda Ingram [25]; "My favorite thing in the world is a box of fine European chocolates which is, for sure, better than sex." - Alicia Silverstone [25]; “All you need is love" (where the word love is crossed out and chocolate written in) - Anonymous [26]; and "Forget love ... I'd rather fall in chocolate!" - Anonymous [25]. Instances like these reprise a theme in which chocolate is compared to (or substituted for) love and sex - with the comparison favoring chocolate - for women (albeit often with humorous intent). This depiction seems quite specific to chocolate among food products and specific to women. Our findings are consistent with but do not compel these characterizations. Indulgence in the putatively preferred comparator (chocolate) might relieve the desire for the supposedly less gratifying substitute (sex).

We do not have access to data on the frequency of sex, and interest in chocolate, to examine the converse relationship.

Mechanism

A biological underpinning for such a proposed explanation is reflected in the inference that “Chocolate gets right to the heart of sexual pleasure by increasing the brain’s level of serotonin” [6]. (Indeed, chocolate does contain phenylethylamine and stimulates biogenic amines, including serotonin and dopamine as well as catecholamines [5].) The differential effects in men vs women could be speculated to align with observations that different brain regions are activated and inhibited by chocolate consumption, and chocolate “satiety,” in women vs men [27].

Limitations

This study has limitations. It is cross-sectional: temporality is not known and causality cannot be inferred. Though it was noted that these findings are consistent with a portrayal of a chocolate-substituting-for-sex-in-women portrayal that is rife in the lay literature, they, by no means, compel that interpretation. Potential for bias and confounding are inherent to observational studies. The study did not include women of childbearing potential, and findings might not extend to this group. However, the relationship was by no means attenuated (indeed, showed a suggestion of being strengthened) for the youngest women among those assessed. The study was relatively generally sampling but did have other exclusions, and findings need not extend to excluded groups such as those with heart disease, diabetes, or cancer. The fact that questions about sex were not a central focus of the parent study, and did not figure in the recruitment process may be a relative strength, reducing participation bias based on the outcome - for a potentially sensitive topic. An additional strength is the large sample size and access to key relevant covariates, including measured testosterone, blood pressure, calorie intake, and mood.

## Conclusions

Women who eat chocolate more often report less interest in sex. Future studies are needed to replicate the finding, extend it to excluded groups, and assess whether the relation of chocolate consumption to sex-interest in women is causal (in the typically represented direction). With the satisfactions of chocolate substituting for - or perhaps surpassing - the fulfillments normally sought from amorous activities, chocolate consumption may (in accordance with popular tropes) temper the drive for sex itself.
